# Psychological interventions for dancers’ mental health: a systematic review

**DOI:** 10.1186/s40359-025-03724-7

**Published:** 2025-11-22

**Authors:** Yunxi Zhang, Young-Eun Noh, Siqi Liu

**Affiliations:** 1 Department of Physical Education, Chang’an University, Xi’an, ShaanXi Province 710064 China; 2 Faculty of Sports and Exercise Science, Universiti Malaya, Kuala Lumpur, 50603 Malaysia

**Keywords:** Performing artist, Meditation-based intervention, Dance injury, Confidence, Body image, Performance anxiety, Psychological rehabilitation, Injury recovery

## Abstract

**Background:**

Despite the high prevalence of mental health challenges among dancers, research on psychological interventions for this population has only recently begun to emerge and has not yet been systematically synthesized.

**Objective:**

This systematic review aimed to identify existing interventions for supporting dancers’ mental health and to analyze their effectiveness, feasibility, and limitations.

**Methods:**

This study followed the Preferred Reporting Items for Systematic Reviews and Meta-Analyses (PRISMA) guidelines.

**Results:**

Eleven studies were included and categorized based on the intervention focus: (1) managing performance anxiety and emotional regulation (*k* = 6), (2) reducing eating disorder symptoms (*k* = 4), and (3) promoting psychological rehabilitation for injured dancers (*k* = 1).

**Conclusion:**

The findings indicated that although various interventions have been implemented to reduce performance anxiety, eating disorder symptoms, and negative psychological responses following injuries, as well as to aid in the management of emotional regulation, their effectiveness remains inconsistent due to differences in intervention type, intervention duration, and participants’ time constraints. Future studies should develop targeted, evidence-based interventions that consider the unique demands of the dance profession.

**Supplementary Information:**

The online version contains supplementary material available at 10.1186/s40359-025-03724-7.

## Introduction

The dance profession requires exceptional discipline, endurance, and emotional expression. Dancers face intense training schedules, constant performance pressure, and high expectations, which place them at risk of both physical and mental strain. Physically, injuries during both training and performance are prevalent, particularly in the ankles, lower back, and toes [1–3]. Mentally, dancers face substantial psychological strain, with 35.4% of 198 American university dancers reporting symptoms of anxiety and depression [4] and 20.8% of 147 German professional dancers experiencing moderate or severe symptoms of depression, generalized anxiety disorder, or disordered eating [5].

Multiple factors contribute to dancers’ mental health challenges. First, the physical demands of dance increase the risk of injury, irrespective of performance level [1, 6–8]. Injuries often result in negative psychological responses, including fear, sadness, stress, and depression [4, 9–12]. In some cases, acute injuries may evolve into chronic conditions [13], further exacerbating mental health issues [14]. Second, the high-pressure environment of competitive dance, marked by performance demands, peer comparison, and internalized expectations, can result in emotional exhaustion, heightened stress, and reduced self-confidence [12, 15–17]. These psychological stressors can impair sleep quality [18, 19], increase injury risk [16], alter pain perception [20], and compromise performance [21]. A 44-week longitudinal study reported that nearly one-third of professional ballet dancers experienced mental health difficulties during the dance season in Germany [22]. Third, body image dissatisfaction significantly contributes to psychological distress among dancers [23–27]. Overemphasizing a lean body type [28] causes many dancers to express dissatisfaction with specific body parts, including the pectoral muscles, thighs, biceps, and abdomen [29], despite having a healthy body mass index. In pursuit of an ideal physique, they may engage in restrictive dieting or weight-control behaviors [30–34]. For example, 18 (47.4%) out of 83 elite ballet dancers from the Paris National Opera were reported to have a history of eating disorders [35]. These behaviors are closely linked to a strong dance identity and are more pronounced among dancers than in nondancer populations [32, 36].

The combination of physical injuries, performance-related stress, and body image dissatisfaction places dancers at elevated risk of psychological distress [37, 38], which not only impairs performance and increases injury risk [4, 22, 39–42] but also diminishes overall well-being [43, 44]. For example, performance anxiety, recognized as a prevalent mental health concern [45], has been associated with maladaptive outcomes such as disordered eating behaviors and mood-related symptoms among dancers [46]. Although some dancers seek social support [12, 17, 43], such coping mechanisms remain insufficient [18].

Psychological interventions are a well-established means of alleviating distress and enhancing mental health in performance-focused populations, including injured athletes [47]. Among these, mindfulness-based approaches have shown particular effectiveness in reducing anxiety and improving emotional regulation. For instance, mindfulness training has been found to reduce performance anxiety in musicians [48], while mindfulness-acceptance-commitment interventions have improved mental health outcomes in professional ballet dancers [49]. Beyond symptom-focused or disorder‑specific approaches, positive psychology interventions that cultivate strengths, gratitude, and mindful awareness have enhanced resilience, stress management, and relational climate among educators in higher education, suggesting complementary pathways to support dancers’ well‑being [50]. These findings suggest promising avenues for dancers; however, the broader landscape remains underexplored. It is still unclear which types of interventions are most effective, for which psychological outcomes, and across which dancer populations. To date, this evidence has not been systematically synthesized. As Podrihalo et al. [51] emphasize, although the prevalence and severity of mental health concerns among dancers are well documented, systematic efforts to identify, evaluate, and implement psychological interventions remain scarce. Without such synthesis, healthcare practitioners and educators lack the evidence-based guidance needed to support dancers’ mental health.

To address this critical gap, this systematic review aimed to identify, evaluate, and synthesize existing intervention strategies targeting mental health issues among dancers in order to inform future research, guide practitioners, and support the development of effective context-specific mental health programs for the dance community. The guiding research question was as follows: What psychological interventions have been developed and evaluated to address mental health challenges in dancers? By systematically identifying and evaluating existing psychological interventions, this review addresses a critical gap in the literature, as no prior synthesis has focused specifically on dancers. This review not only highlights effective and promising approaches but also provides evidence-based guidance for practitioners and educators. Such guidance is essential for supporting dancers’ psychological well-being amid the unique cultural and performance demands of dance.

## Methods

### Study design

Given that this review included both qualitative and quantitative designs, we followed methodological guidance for systematic mixed studies reviews [52] and applied the Mixed Methods Appraisal Tool (MMAT, version 2018) to assess study quality. This review was not preregistered. To ensure transparency, the Preferred Reporting Items for Systematic Reviews and Meta-Analyses (PRISMA) checklist [53] was included to demonstrate adherence to reporting guidelines (see Supplementary Material 1). The synthesis that follows integrates the available evidence on psychological interventions for dancers.

### Eligibility criteria

In line with the World Health Organization [54], mental health was operationally defined as a continuum of mental well-being, ranging from positive outcomes (e.g., resilience, coping, or well-being) to difficulties such as anxiety, depression, stress, eating disorders, or burnout. Studies were included if (1) they were published in English, (2) they described an intervention aimed at improving participants’ mental health, including any aspects of psychological well-being identified in previous literature as relevant to dancers [37], (3) they assessed the feasibility, acceptability, and practicality of such interventions, and (4) they targeted dancers engaged in artistic, educational, recreational, or competitive dance settings (ballet, contemporary dance, traditional/folk dance, hip-hop dance, ballroom dance, modern dance, etc.). These dance types were considered within broader cultural, performance, and training contexts that are more representative of general dance populations.

Studies were excluded if (1) they focused on physiological disorders (e.g., epilepsy), (2) they were protocol studies that only described the study design, methodology, and expected outcomes without providing actual data or research findings, (3) they were nonexperimental, including those evaluating previous interventions, or (4) they focused on dance forms primarily performed in adult-entertainment or occupational contexts (e.g., exotic dancing, bar dancing, and other performance settings in which sexualized labor is central). These dance forms were excluded because of the distinct sociocultural, occupational, and psychological challenges associated with them (often involving complex intersections of gender, sexuality, stigma, labor dynamics, objectification, social marginalization, and identity negotiation), which differ significantly from those experienced in more conventional artistic, educational, recreational, or athletic dance settings [55]. Including such studies would have introduced substantial heterogeneity and potentially obscured the specific mental health patterns relevant to the general dance population.

### Search strategy

Two methods were employed to identify relevant studies. First, we conducted systematic searches in five electronic databases: PubMed, Psychology & Behavioral Sciences Collection, SPORTDiscus, Web of Science, and Scopus. Searches were performed in the title and abstract fields for PubMed and Psychology & Behavioral Sciences Collection, and in the title, abstract, and keyword fields for Web of Science, Scopus, and SPORTDiscus. The strategy used a Boolean combination of two blocks: (1) dance-related terms and (2) intervention/mental health-related terms. The detailed terms for each block are presented in Table 1. All databases were searched from inception through May 14, 2025, using the same set of reported keywords.

**Table 1:** Search strategy used across databases

Block	Keywords
Dance-Related Terms	danc* OR ballet* OR jazz* OR choreograph* OR “perform* art*”
Intervention/Mental Health-Related Terms	project* OR intervention* OR “mental training” OR “mental skill*” OR “psychological skill*” OR “psychological training” OR mindfulness OR meditation* OR CBT OR resilien* OR “coping skill*” OR “mental health” OR well-being OR therap* OR yoga* OR anxi* OR depression OR distress* OR stress* OR PTSD OR disorder* OR nervosa* OR insomnia* OR panic* OR emotion* OR “body image” OR self-esteem* OR burnout*
Combined Search	Block (1) AND Block (2)
Search Fields	(a) PubMed and Psychology & Behavioral Sciences Collection: Title and Abstract (b) Web of Science, Scopus, and SPORTDiscus: Title, Abstract, and Keywords

Second, to enhance coverage, we also employed manual searches, including forward citation tracking of references from the electronic database search as well as forward and backward citation tracking of existing scoping reviews on dancers’ mental health [37]. The database and manual searches were conducted by the first author.

### Study screening and selection

Duplicate records were removed using EndNote version 20 (Clarivate Analytics). Titles and abstracts were then screened, followed by a full-text assessment of potentially eligible studies. Two reviewers (the first and third authors) independently screened the titles and abstracts of all identified studies, achieving a high level of agreement (> 85%). The same reviewers independently conducted full-text screening, with similarly high agreement (> 85%). Any discrepancies at both stages were discussed and resolved through consensus.

### Data extraction

The first author extracted data from the selected studies into Microsoft Excel, documenting the study authors, publication year, intervention characteristics (e.g., research design, type of intervention, modality, dosage, and implementer), participant information (e.g., sample size, gender, type of dance, and age), assessment characteristics (e.g., measurement tools, timing of assessments, and adherence), type of analysis, effect sizes, and main results. Details regarding the timing of assessments (e.g., baseline, post-intervention, and follow-up) and participant adherence or attrition, including reasons for dropout where available, were recorded. The third author cross-checked the extracted data. Discrepancies were resolved through discussion among all authors.

### Data analysis

Data from the included studies were analyzed using a narrative synthesis approach [56], integrating evidence from qualitative, quantitative, and mixed-methods research. The studies were summarized descriptively and organized according to the mental health outcome under focus. Key outcomes, intervention characteristics, and participant information were then extracted and compared across studies to provide a comprehensive overview of both the effectiveness and characteristics of the interventions targeting dancers’ mental health.

### Quality assessment

We used the MMAT [52] to assess the quality of included studies, as this assessment tool includes criteria for five research design types (i.e., qualitative, randomized controlled, nonrandomized, quantitative descriptive, and mixed methods). This tool is widely used in systematic reviews that synthesize heterogeneous evidence [52]. Each criterion includes three options: “Yes,” “No,” or “Can’t tell.” The first and third authors independently assessed study quality using the MMAT, achieving high inter-rater agreement with Cohen’s kappa (k = 0.82). Discrepancies were resolved after discussion until a consensus was reached.

## Results

### Study selection and characteristics

**Fig. 1:**
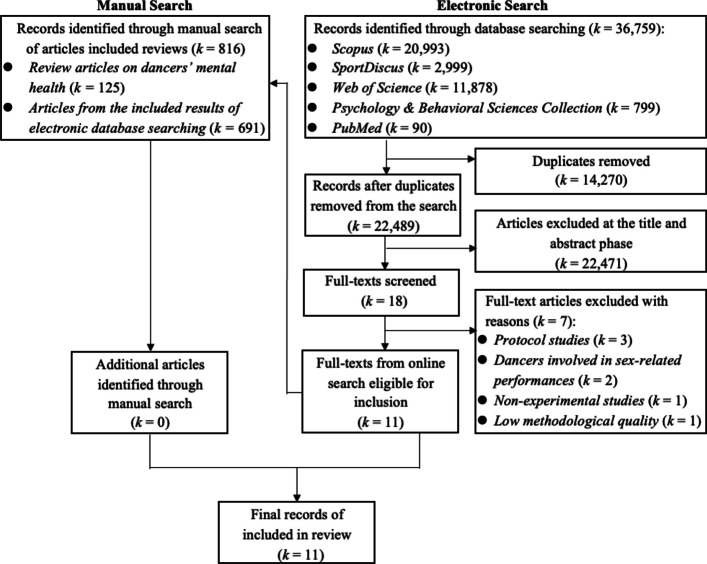
PRISMA flow diagram

The electronic search and manual search yielded 36,759 and 816 articles, respectively (see Fig. 1). Following the screening and eligibility assessment, 11 studies were included in this review, comprising a total of 234 dancers, with individual sample sizes ranging from 1 to 48 participants. Overall, female dancers comprised the majority of participants, accounting for 75.6% of the total sample across all included studies. One study did not specify the type of dancer involved [57], while the others focused on contemporary dancers [58], auxiliary dancers [59], classical dancers [60], a solo dancer [61], ballet dancers [49, 62–64], and Latin dancers [65]. Participants ranged in age from approximately 14 to 43 years, and their skill levels varied from recreational [64] to professional [49, 61, 62].

The 11 included studies were conducted across eight countries, with the largest representation from the United States (*k* = 3), followed by Australia (*k* = 2), China (*k* = 1), Austria (*k* = 1), Mexico (*k* = 1), Switzerland (*k* = 1), Sweden (*k* = 1), and the United Kingdom (*k* = 1). In terms of study design, pre- and post-tests were most common (*k* = 5), followed by randomized controlled trials (RCTs) (*k* = 2), nonrandomized trials (*k* = 2), pilot studies (*k* = 1), and a single-case study (*k* = 1).

The included studies were published between 2011 and 2025, spanning 14 years. Strikingly, 9 of the 11 studies were published within the last three years (2023–2025), reflecting a sharp increase in scholarly attention to dancers’ mental health interventions and suggesting that this is an emerging and rapidly developing area of research. However, the concentration of studies in such a short time frame also indicates that the field is still in its infancy, with limited opportunities for replication, longitudinal evaluation, or systematic accumulation of evidence. This highlights both the timeliness and the fragility of current findings, underscoring the need for sustained research efforts to build a more robust evidence base.

Based on the mental health focus, studies were categorized into (1) interventions for managing performance anxiety and emotional regulation (*k* = 6), (2) interventions for reducing eating disorder symptoms (*k* = 4), and (3) interventions involving psychological rehabilitation for injured dancers (*k* = 1). In terms of intervention lengths, aside from one study that reported five 90-min sessions without specifying the total number of weeks, the remaining ten studies could be grouped into three categories: short-term (1–4 weeks; *k* = 4), medium-term (5–8 weeks; *k* = 4), and long-term (10–12 weeks; *k* = 2). The results of the summary data are presented in Table 2.

**Table 2:** Summary of key findings from the included studies (Chronological Order)

Author(s) and year	Aim	Intervention				Participants				Assessment characteristics			Type of analysis	Effect sizes (as reported)	Main results
		**Design**	**Type of intervention**	**Modality and dosage**	**Implementer**	**Sample size**	**Sex**	**Type of dance**	**Age** (*M* ± *SD*)	**Measurement tools**	**Timing of assessments**	**Adherence**			
	(1) Interventions for Managing Performance Anxiety and Emotional Regulation														
Walton et al., 2025 [57]	• To test the acceptability, feasibility, and effectiveness of a brief compassion-based intervention on performing artists • To explore the participants’ perspectives about the further development of compassion-based interventions	Pre-post test	Self-compassion meditation intervention	• Online • 3 weeks • 10 min per session • Once a day	None	Performing artists (*N* = 10) • Music (*n* = 5) • Acting (*n* = 3) • Dance (*n* = 2)	• Male (*n* = 3) • Female (*n* = 6) • Binary individual (*n* = 1)	• Dancers (*n* = 2) – No specific dance type was mentioned	43.5 ± 12.6	• Compassionate Motivation and Action Scale (CMAS-SC) • Self-Compassion Scale (SCS) • Fears of Self- Compassion Scale (FSCS) • Warwick-Edinburgh Mental Wellbeing Scale (WEMWBS) • Patient Health Questionnaire-9 (PHQ9) Generalized Anxiety Disorder-7 (GAD7) • Perceived Stress Scale (PSS) • Body Appreciation Scale-2 (BAS2) • Alcohol Use Disorders Identification Test (AUDIT-C) • Depression, Anxiety and Stress Scales (DASS-21) • Implementation Outcome Measures	• Baseline (t0): Pre-intervention • Post-intervention (t1): Week 3)	100% completed	• Pearson correlation analysis • Reflexive thematic analysis	CMAS (Cohen’s *d* = 0.73)—medium SCS (Cohen’s *d* = 0.74) – medium FSCS (Cohen’s *d* = −0.40) – small WEMWBS (Cohen’s *d* = 0.25) – small DASS-21 (Cohen’s *d* =—0.51) – medium BAS2 Cohen’s *d* =—0.34)—small	• The improvement in self-reported mental health scale scores suggested good acceptability, appropriateness, and feasibility • After receiving the compassion-based interventions, participants self-reported an increase in well-being and a reduction in psychological distress
Chen et al., 2024 [64]	To evaluate the effect of systematic desensitization training on competition anxiety in the training of Latin dancers	Non-RTC	Systematic desensitization training (including imagery desensitization and in vivo desensitization)	• Offline • 8 weeks • Twice a week (The duration of each intervention was not provided)	Instructor	*N* = 48	• Male (*n* = 24) • Female (*n* = 24)	Latin dancers (*N* = 48)	Between 23 to 25 years old	• Sports Competition Anxiety Test Questionnaire • Sport Competition Trait Anxiety Inventory • Competitive State Anxiety Inventory	• Baseline (t0): First week of intervention • Post-intervention (t1): Week 8	100% completed	One-way ANOVA	Not reported	• The systematic desensitization training can significantly diminish competitive anxiety among Latin dancers
Harrison et al., 2025 [49]	To investigate the effectiveness of the Mindfulness-Acceptance-Commitment intervention for professional ballet dancers	RTC	Mindfulness-acceptance-commitment intervention	• Offline • 6 weeks • 30 min per session • Once a week	An accredited instructor	*N* = 16	• Male (*n* = 2) • Female (*n* = 14)	Professional ballet dancers (*N* = 16)	25 ± 4.88	• The Mindfulness Inventory for Sport • The Acceptance Action Questionnaire	• Baseline (t0): First week of intervention • Post-intervention (t1): week 6	100% completed	• One-way ANOVA • Thematic analysis	Not reported	• The Mindfulness-Acceptance-Commitment intervention enhanced participants awareness of mental health, alleviated performance-related tension and anxiety, improved focus during performances, and reduced stress
Lubert et al., 2023 [58]	To examine the effects of psychological choking interventions tailored to “choking-susceptible” performing artists individually in a coaching setting	Pre-post test	Tailored Choking Intervention (including acclimatization training, goal setting, imagery, self-talk, and relaxation techniques)	• Offline • 10 weeks • Every two weeks • 54 ± 4.9 min per session	A certified psychologist	Performing artists (*N* = 9) • Musician (*n* = 6) • Dancers (*n* = 2) Actress (*n* = 1)	• Male (*n* = 1) • Female (*n* = 8)	Contemporary dancers (*n* = 2)	23.3 ± 2.2	• A Short Version of the Kenny Music Performance Anxiety Inventory • The Brief Fear of Negative Evaluation Scale-Revised • General Self-Efficacy Scale • Mental Readiness Form-3	• Baseline (t0): First week of intervention • Post-intervention (T1): week 10	90% completed (refusing to receive the intervention [*n* = 1])	• T-test • Reflexive thematic analysis	Trait performance anxiety (Cohen’s *dz* = 0.96) – large Fear of negative evaluation (Cohen’s *dz* = 1.28) – large Self-efficacy (Cohen’s *dz* = 1.02) – large Cognitive anxiety (Cohen’s *dz* = 1.91) – large Somatic anxiety (Cohen’s *dz* = 1.09) – large Confidence (Cohen’s *dz* = 1.38) – large Heart rate (Cohen’s *dz* = 0.46) – small Performance quality (Cohen’s *dz* = 0.93)—large	• Quantitative data showed reductions in performance anxiety and fear of negative evaluation, and increases in self-efficacy and performance quality, from before to after the intervention phase • Themes from qualitative analysis comprised managing nervousness and feeling more relaxed, becoming more self-confident, satisfaction with artistic and mental performance, feeling good and enjoying performing, and general positive effects
Stackpole and Quiroga-Garza, 2023 [60]	To test the effectiveness of an intervention based on solution-focused brief therapy for dance students, specifically whether it can improve their performance and reduce anxiety levels	Pre-post test	A Solution-Focused Brief Therapy	• Offline • 5 sessions • 90 min per session	Research team members	*N* = 16	• Male (*n* = 6) • Female (*n* = 12)	• Contemporary dancers (*n* = 8) • Classical dancers (*n* = 8)	17.81 ± 3.14	• Kenny Music Performance Anxiety Inventory • Believability of Anxious Feelings and Thoughts Questionnaire • Self-perceived Anxiety Evaluation	• Baseline (t0): First session of intervention • Post-intervention (t1): 4 months later	100% completed	• Correlation analysis • Wilcoxon signed-rank test • Thematic analysis	Not reported	• The solution-focused brief therapy can be a valuable tool for dance teachers looking to enhance the performance of their students, reduce their anxiety levels to overcome stage fears, and improve their overall performance
Wallman-Jones et al., 2023 [64]	To explore the feasibility of an eight-week Feldenkrais Method intervention to enhance interoceptive processes and psychological well-being in a population of adolescent female recreational ballet dancers	Pre-post test	Feldenkrais Method	• Offline • 8 weeks • 60 min per session • Once a week	A certified Feldenkrais Method practitioner	*N* = 12	Female (*N* = 12)	Recreational-level adolescent ballet dancers (*N* = 12)	14.25 ± 1.29	• Interoceptive Accuracy • Multidimensional Assessment of Interoceptive Awareness • Basic Psychological Need Satisfaction and Frustration Scale	• Baseline (t0): Pre-intervention • Post-intervention (t1): Week 8	100% completed	• Wilcoxon Signed-Rank test • Mann–Whitney U test • Thematic analysis	Not reported	The study preliminarily demonstrated the feasibility of the Feldenkrais Method in adolescent female recreational ballet dancers, showing improvements in attention regulation and reduced social comparison, while its effects on other psychological well-being and interoceptive ability indicators remain unclear, requiring further research for validation
	(2) Interventions for Reducing Eating Disorder Symptoms														
Gorrell et al., 2024 [66]	• To test the subsequent adaptation, the athlete's body project, tailored for younger dancers • To evaluate the preliminary efficacy of the athlete body project in reducing eating disorder symptoms, drive for muscularity, and in improving body appreciation both immediately post-program, as well as at 6–8-week follow-up	Pilot Study	Athlete body project	• Offline • 3 weeks • 120 min per session • Once a week	One group leader, along with one to two peer assistants	*N* = 23	• Male (*n* = 12) • Female (*n* = 11)	Teenage ballet students (*N* = 23)	• School A (girls: 16.5 ± 0.84; boys: 16.83 ± 0.75) • School B (girls: 14.17 ± 0.41; boys: 13.8 ± 0.45)	• Eating Disorders Examination—Questionnaire (EDE-Q) • Body Appreciation Scale (BAS) • Drive for Muscularity Scale (DMS)	• Baseline (T0): Before program start • Post-program (T1): Week 3 • Follow up: (T2): 6–8 weeks after program completion	• T0: 100%, • T1: 57–61% (Not included due to lack of consent [*n* = 9] and incomplete baseline questionnaires [*n* = 2]) • T2: 43–52% (incomplete baseline questionnaires [*n* = 11 or 12])	• Paired-samples *t*-test • One-way ANCOVA	EDE-Q: T2 (*d* = 0.14)—small, T3 (*d* = 0.02) BAS: T2 (*d* = 0.39), T3 (*d* = − 0.21) – small DMS: T2 (*d* = 0.12), T3 (*d* = 1.24)—large T1 = baseline T2 = post-program T3 = follow-up	• The program met pre-determined benchmarks for feasibility and acceptability • This non-randomized pilot trial demonstrated non-significant reductions in eating pathology, non-significant increases in body appreciation, and significant reductions in drive for muscularity
Gorrell et al., 2021 [62]	• To assess the acceptability and feasibility of the adaptation of a healthy living intervention within a sample of elite, professional ballerina • To evaluate the preliminary effectiveness of an adapted female athlete body project intervention in reducing body dissatisfaction, dietary restraint, and eating disorder symptoms both immediately post-intervention, as well as at six-week follow-up	RTC	An adaptation of a healthy living intervention	• Offline • 3 weeks • 120 min per session • Once a week	One group leader, along with one to two peer assistants	*N* = 19	Female (*N* = 19)	Professional ballet dancers (*N* = 19)	23.21 ± 5.75	• Dutch Restrained Eating Scale • Eating Disorders Examination Questionnaire	• Baseline (T0): Before program start • Post-program (T1): Week 3 • Follow up: (T2): 6 weeks after program completion	100% completed	• Mann–Whitney U test • Wilcoxon signed-rank test • Friedman’s ANOVA	Not reported	• The acceptability of this program appears to be robust • Some modifications (i.e., adjusting recruitment strategies, increasing administrative involvement, and enhancing the role of peer leaders) have the potential to increase the feasibility of this intervention • Dancers in the intervention program demonstrated significant decreases in reported body dissatisfaction, dietary restraint, and a global measure of eating disorder pathology following the intervention. These findings were maintained at the six-week follow-up assessment, suggesting that the potential beneficial effects of the intervention may be sustainable over time
Nordin-Bates et al., 2023 [63]	To evaluate effects of a combined cognitive behaviour therapy and nutrition education intervention on symptoms, risk factors (perfectionism), and potential protective factors (self-esteem, self-compassion) for disordered eating behaviors in 12–15-year-old dancers	Pre-post test	A combined cognitive behaviour therapy and nutrition education intervention	• Offline • 8 weeks • Cognitive behaviour therapy workshop: 5 sessions; 90 min per session • Nutrition workshops: 4 sessions; 45 min per session	Instructor	*N* = 40	• Male (*n* = 9) • Female (*n* = 31)	Teenage ballet students (*N* = 40)	± 0.84	• Swedish version of the Rosenberg Self-Esteem Scale • Swedish version of the short form of the Self- Compassion Scale • Multidimensional Perfectionism Scale • Multidimensional Inventory of Perfectionism in Sport • Swedish version of the Eating Disorder Examination Questionnaire	• Baseline (T0): Before phase 1 • After phase 1 (T1): week 8 • Before phase 2: (T2): Week 8 • After phase 1 (T3): Week 16	T0: 100%, T1:100%, T2: 62.5% T3: 62.5% (reasons for attrition not reported)	• Pearson correlation analysis • Friedman test	Kendall’s W (.02—.08), except for self-esteem where W =.20; a small effect	• The intervention did not yield any discernible impact on the symptoms for disordered eating behaviours
Torres-McGehee et al., 2011 [59]	To test the efficacy of a team-centered program to prevent characteristics and behaviors related to eating disorders (e.g., body dissatisfaction, drive for thinness, self-esteem, bulimia attitudes, perfectionism, maturity fears, and depression) in at-risk, previously under-studied collegiate auxiliary dancers	Non-RTC	A team-centered educational program (including curricula on sports nutrition, mental health, and a balanced discussion of the consequences of substance use and other unhealthy behaviors)	• Offline • 4 weeks • 45 min per session • Twice a week	Peer leaders and research team members	*N* = 40	Female (*n* = 40)	Collegiate auxiliary dancers (*N* = 40)	• Intervention group: 19.2 ± 1.2 • Control group: 19.1 ± 1.0	• Eating Disorder Inventory-3 • Center for Epidemiological Studies Depression Scale • Eating Disorder Knowledge Questionnaire • A nutritional knowledge survey specific to athletes and dancers	• Baseline (T0): Pre-intervention • Post-intervention (T1): Week 4	95% (not completing the intervention [*n* = 2])	• Independent-samples *t*-test • ANCOVA	Not reported	The team-centered intervention effectively improved collegiate auxiliary dancers’ knowledge and attitudes toward eating disorders, supporting its feasibility and impact in this population
	(3) Interventions for Psychological Rehabilitation in Injured Dancers														
Roncaglia, 2023 [61]	To present and reflect on the lessons learnt from practice with an individual case professional dancer who sought comprehensive psychological support as a result of a sustained trauma-injury, ruptured Achilles tendon during a live performance	Single case	A psychological support program	• Offline • 12 weeks • 60 min per session • Once a week	The clinicians	*N* = 1	Male (*N* = 1)	A professional senior soloist dancer (*N* = 1)	Not provided	Positive and Negative Affect Schedule	Not applicable	100% completed	Reflective thematic analysis	Not reported	Through reflective practice, three main themes emerged as acquired skills through the psychological interventions offered: 1) self-confidence and self-esteem, 2) a renewed sense of belonging, and 3) a sense of autonomy

*RTC* Randomized controlled trial

Among the studies included, only Walton et al. [57] met all five MMAT criteria and was rated as high quality (see Table 3). Of the remaining 10 studies, based on the number of MMAT criteria satisfied, three were rated as upper-middle quality [49, 60, 61], three as moderate quality [59, 64, 65], two as lower-middle quality [62, 66], and one as low quality [58]. The detailed MMAT appraisal, together with accompanying comments, is presented in Supplementary Material 2.

**Table 3:** MMAT quality appraisal of studies (Chronological Order)

Author(s)	Screening Questions		Qualitative					Quantitative (randomized)					Quantitative (non-randomized)					Mixed Methods				
	Q1	Q2																				
Walton et al. (2025) [57]	✓	✓																✓	✓	✓	✓	✓
Harrison et al. (2025) [49]	✓	✓						✓	✓	✓	–	✓										
Chen et al. (2024) [65]	✓	✓											✓	✓	X	✓	–					
Gorrell et al. (2024) [66]	✓	✓											X	X	✓	X	✓					
Lubert et al. (2023) [58]	✓	✓											X	X	X	✓	X					
Nordin-Bates et al. (2023) [63]	✓	✓											✓	✓	✓	✓	X					
Roncaglia (2023) [61]	✓	✓																				
Stackpole & Quiroga-Garza (2023) [60]	✓	✓											✓	✓	X	✓	✓					
Wallman-Jones et al. (2023) [64]	✓	✓	✓	X	X	✓	✓															
Gorrell et al. (2021) [62]	✓	✓						✓	X	X	X	✓										
Torres-McGehee et al. (2011) [59]	✓	✓											✓	✓	X	X	✓					

Q1: Are there clear research questions? Q2: Do the collected data allow addressing the research questions?

‘✓’ means that the criterion is met. ‘X’ means that the criterion is not met. ‘–’ means that there is not enough information in the paper to judge if the criterion is met or not. Five criteria for the qualitative studies are as follows: 1. Is the qualitative approach appropriate to answer the research question? 2. Are the qualitative data collection methods adequate to address the research question? 3. Are the findings adequately derived from the data? 4. Is the interpretation of results sufficiently substantiated by data? 5. Is there coherence between qualitative data sources, collection, analysis and interpretation?

Five criteria for the quantitative (randomized) studies are as follows: 1. Is randomization appropriately performed? 2. Are the groups comparable at baseline? 3. Are there complete outcome data? 4. Are outcome assessors blinded to the intervention provided? 5. Did the participants adhere to the assigned intervention?

Five criteria for the quantitative (non-randomized) studies are as follows: 1. Are the participants representative of the target population? 2. Are the measurements appropriate in relation to both the outcome and the intervention (or exposure)? 3. Are the outcome data complete? 4. Are the confounders addressed in the study design and analysis? 5. Was the intervention administered (or exposure occurred) as intended during the study period?

Five criteria for the mixed methods studies are as follows: 1. Is there an adequate rationale for using a mixed methods design to address the research question? 2. Are the different components of the study effectively integrated to answer the research question? 3. Are the outputs of the integration of qualitative and quantitative components adequately interpreted? 4. Are divergences and inconsistencies between quantitative and qualitative results adequately addressed? 5. Do the different components of the study adhere to the quality criteria of each tradition of the methods involved?

Since Quantitative (descriptive) studies were not included, they are not displayed in the table.

### Interventions for managing performance anxiety and emotional regulation

Across the six studies targeting performance anxiety and emotional regulation [49, 57, 58, 60, 64, 65], a range of psychological approaches (e.g., mindfulness, compassion-based meditation, the Feldenkrais method, solution-focused therapy, and systematic desensitization) were implemented with dancers from diverse backgrounds. Despite the methodological heterogeneity, the overall findings suggested that these interventions can reduce performance-related anxiety, improve attentional control, and enhance well-being. Several studies also reported gains in self-efficacy, confidence, and focus during performance, although the effects on broader psychological outcomes (e.g., body awareness) were less consistent. Notably, most interventions were conducted with small samples and varied considerably in terms of format, dosage, and delivery, which limited comparability; however, collectively, they demonstrated the promise of tailored psychological strategies for managing performance anxiety among dancers. While the overall evidence suggested promising effects, the specific approaches and outcomes varied across studies, as outlined below.

Lubert et al. [58] conducted a 10-week tailored choking intervention (including acclimatization training, goal setting, imagery, self-talk, and relaxation techniques), followed by semi-structured interviews with nine performing artists (i.e., six musicians, two dancers, and one actress) in Austria. Qualitative data indicated that this intervention helped participants better manage competitive anxiety, increased their confidence, enhanced their performance experience and satisfaction, and improved their dance performance. Moreover, a quantitative assessment indicated that the intervention reduced performance anxiety (*d*_*z*_ = 0.96) and fear of negative evaluation (*d*_*z*_ = 1.28) and increased self-efficacy (*d*_*z*_ = 1.02) and performance quality (*d*_*z*_ = 0.93). The reported effect sizes (*d*_*z*_ = 0.93–1.28) are all large, suggesting that the intervention had a substantial positive impact across multiple performance-related outcomes. Taken together, these findings imply that the intervention not only reduced maladaptive psychological processes but also enhanced adaptive ones, leading to both mental and performance-related benefits.

Similarly, Walton et al. [57] surveyed performing artists in Australia, including actors, musicians, and dancers, to examine the relationship between self-compassion and mental health. They found that dancers reported higher levels of stress, anxiety, and depression than actors and musicians. Based on these findings, the researchers then invited back a nonrandom group of 10 performing artists to complete a compassion-based meditation intervention. The intervention involved providing participants with links to MP3 files containing guided compassion meditations, each approximately 10 min long. Participants were required to practice one type of meditation daily over 3 weeks. Following 21 sessions of compassion-based meditation, participants demonstrated medium-to-large improvements in self-compassion (*d* = 0.74), alongside small but positive improvements in well-being (*d* = 0.25) and body appreciation (*d* = 0.34). These results indicate that the intervention was particularly effective in enhancing self-compassion, a core protective factor for dancer populations, while also contributing modest gains in broader outcomes such as well-being and body appreciation. Taken together, the findings highlight the preliminary promise of this approach and underscore the need for further investigation with larger samples.

Apart from the two studies [57, 58] examining performing artists more broadly, four studies focused exclusively on dancers, including ballet, Latin, classical, and contemporary dancers [49, 60, 64, 65]. Harrison et al. [49] conducted a 6-week mindfulness–acceptance–commitment intervention with 16 professional ballet dancers in Australia. Each 30-min session was conducted under the guidance of an accredited instructor and incorporated audio mindfulness practical exercises. Post-intervention interviews revealed that the mindfulness intervention enhanced participants’ mental health awareness and alleviated their performance-related tension, anxiety, and stress, while improving focus during performance.

Similarly, addressing performance anxiety among dancers, Stackpole and Quiroga-Garza [60] conducted five brief solution-focused therapy sessions with 16 classical and contemporary dancers from a dance institute in Mexico. Four months post-intervention, participants exhibited reductions in performance anxiety and self-perceived anxiety levels. Moreover, compared with the previous two studies that recruited 16 participants each, Chen et al. [65] recruited a larger sample of 150 dancers in China for an intervention targeting performance anxiety, from which the 48 participants with the highest anxiety scores were selected to complete a systematic desensitization program (intervention group: *n* = 24; control group: *n* = 24), including imagery desensitization and in vivo exposure. Post-intervention results indicated significant reductions in competitive anxiety in the intervention group. Collectively, these three studies [49, 60, 65] demonstrate that different types of psychological interventions (e.g., mindfulness–acceptance–commitment intervention, solution-focused therapy, and systematic desensitization training) can effectively reduce performance anxiety among dancers.

Unlike Harrison et al. [49], who focused on professional ballet dancers, Wallman-Jones et al. [64] targeted the mental health of adolescent ballet dancers in Switzerland. Specifically, they evaluated the effectiveness of an 8-week intervention for 12 female adolescent ballet dancers using the Feldenkrais method, which is a movement therapy intended to improve body movement and psychological well-being by reorganizing connections between the brain and the body. The intervention group (*n* = 6) engaged in one additional hour of Feldenkrais method training after their regular weekly ballet practice. The training was delivered in a group setting in which a certified Feldenkrais practitioner provided verbal guidance to lead students through several functionally relevant movement explorations aimed at enhancing sensory awareness, regulating movement range and effort, and discovering new movement patterns. The findings demonstrated the benefits of using the Feldenkrais method for recreational-level adolescent ballet dancers, who showed improvements in attention regulation and reductions in social comparison. However, its impact on broader psychological outcomes and internal body awareness remained uncertain.

### Interventions for reducing eating disorder symptoms

Four studies examined interventions aimed at reducing eating disorder symptoms among dancers, with three focusing on ballet populations [62, 63, 66] and one on auxiliary dancers [59]. Across these studies, the approaches ranged from cognitive behavioral therapy and psychoeducational programs to adapted athlete-focused protocols. While some interventions showed positive outcomes, such as reductions in disordered eating pathology, improved knowledge, and healthier attitudes toward body image, others reported null findings, particularly in adolescent samples. Overall, the evidence suggested that dancer-adapted interventions hold promise, but the results remain inconsistent and limited as a result of small samples, brief intervention durations, and a lack of long-term follow-up. The individual studies are outlined below.

Gorrell et al. [62] implemented a tailored healthy living intervention integrating cognitive behavioral therapy, psychoeducation, and behavioral change strategies among 19 professional ballet dancers in the USA. The intervention lasted for three consecutive weeks, with weekly 1.5-h sessions. After the intervention, participants reported decreases in dietary restraint and overall eating disorder pathology. These improvements were maintained at the 6-week follow-up, suggesting that the beneficial effects of the dancer-adapted intervention may be sustained over time.

Subsequently, Gorrell et al. [66] evaluated the efficacy of another intervention, the Athlete Body Project, in addressing eating disorder symptoms, body appreciation, and drive for muscularity among ballet dancers in the USA. This intervention also comprised a three-week program with weekly 1.5-h sessions, and outcomes were assessed immediately post-intervention as well as at a six- to eight-week follow-up. However, unlike the tailored healthy living intervention for professional ballet dancers reported by Gorrell et al. [62], the pilot trial of the Athlete Body Project did not yield significant results. For eating disorders, small effect at post-program (*d* = 0.14) and virtually no effect at follow-up (*d* = 0.02). This suggests the intervention had minimal impact on disordered eating symptoms, and any slight improvement immediately after the program was not sustained. For body appreciation, a small positive effect at post-program (*d* = 0.39), but it turned negative at follow-up (*d* = − 0.21). For the drive for muscularity, very small effect at post-program (*d* = 0.12) but a large effect on follow-up (*d* = 1.24). This suggests that the intervention may have had unintended or delayed effects, potentially reinforcing muscularity-focused ideals instead of reducing appearance-related pressures. The findings indicate that the intervention had limited immediate benefits for eating disorder symptoms and body appreciation, and in some areas, effects were not sustained. The unexpected large increase in drive for muscularity at follow-up is especially notable and may reflect either a shift in participants’ body ideals (e.g., from thinness to muscularity) or unintended consequences of the program content. This highlights the importance of longitudinal monitoring and suggests that interventions should be carefully tailored to avoid reinforcing alternative body image pressures.

Nordin-Bates et al. [63] administered an 8-week intervention combining cognitive behavioral therapy and nutrition education to 40 teenage ballet students in Sweden. This cognitive behavioral therapy intervention consisted of five sessions based on the “Cool Kids” treatment protocol for adolescent anxiety. It also incorporated elements from acceptance and commitment therapy, focusing on body image and values, as well as compassion-focused therapy. The five sessions included introduction and basic functional analysis (Session 1), goal setting (Session 2), body image (Session 3), emotion regulation (Session 4), and sustainability (i.e., acting in one’s valued direction) (Session 5). These sessions aimed to encourage participants to work toward meaningful personal goals and to cultivate self-compassion. However, the findings indicate that the intervention did not produce measurable changes in perfectionism, self-esteem, self-compassion, or disordered eating symptoms. Across both the control and intervention phases, none of the dependent variables showed statistically significant differences, with p-values ranging from 0.09 to 0.86. The effect sizes were negligible (Kendall’s *W* = 0.02–0.08), suggesting minimal practical impact. The only exception was self-esteem, which showed a small effect (*W* = 0.20), though this did not reach statistical significance. Taken together, these results suggest that the intervention, as implemented, had limited efficacy in modifying key psychological constructs in the sample. This lack of effect may reflect insufficient intervention intensity or duration, or the use of outcome measures that were not sensitive enough to detect subtle changes, thereby reducing the potential for observable improvements.

Compared with the five-session intervention by Nordin-Bates et al. [63], Torres-McGehee et al. [59] extended the program to eight sessions, each lasting 45 min, targeting collegiate female auxiliary dancers in the USA. The sessions covered topics such as sports nutrition and mental health, with a balanced discussion of the consequences of substance use and other unhealthy behaviors. Specifically, they adapted a team-centered eating disorder educational program originally designed for high school athletes for use with collegiate female auxiliary dancers. This intervention effectively improved dancers’ knowledge and attitudes regarding eating disorders and reduced body dissatisfaction and drive for thinness.

### Psychological rehabilitation interventions for injured dancers

Evidence on psychological rehabilitation interventions for injured dancers is extremely limited. Only one single-case study was identified, which described a dancer-centered therapeutic program following a severe injury. Roncaglia, a clinical psychologist in the UK, shared a psychological therapy case involving a male professional senior solo dancer who sustained a ruptured Achilles tendon during a live performance and subsequently underwent surgery [61]. His postoperative psychological rehabilitation was conducted by the clinical psychologist through a 12-session support program. Each session focused on specific themes, including (1) authenticity, openness, and active listening, (2) joint goal setting, process goals, and task goals, (3) congruence, feedback, values, and beliefs, and (4) autonomy, belonging, and competence. Each session lasted 60 min and was held weekly on Saturdays. This dancer-centered psychological intervention helped the dancer regain his confidence and self-esteem, develop a new sense of belonging, and enhance his autonomy.

## Discussion

This review identified and synthesized existing intervention strategies designed to address mental health issues among dancers. Overall, the evidence base for mental health interventions in dancers is limited and of variable quality. Based on the current studies, the certainty of evidence is low to moderate for short-term reductions in performance or competitive anxiety, low for interventions targeting eating disorder symptoms due to mixed findings and methodological limitations, and very low for psychological rehabilitation after injury, as conclusions rely solely on a single-case study.

### Interventions for managing performance anxiety and emotional regulation

More than half of the studies (six out of 11) included in this review focused on interventions addressing performance anxiety and emotional regulation [49, 57, 58, 60, 64, 65]. While mindfulness-based interventions showed preliminary effectiveness for anxiety and emotional regulation, their feasibility remains questionable.

In Walton et al.’s [57] study, despite each meditation practice lasting only 10 min, participants noted that the requirement for daily meditation conflicted with their demanding dance training and competition schedules. The difficulty dancers experienced in adhering to daily or long-duration practices highlights a tension between evidence-based protocols and the lived realities of intensive dance training. These delivery challenges highlight structural barriers in the dance environment. Dancers often juggle multiple rehearsals and performance obligations, suggesting that interventions should be brief, flexible, and seamlessly integrated into daily routines. Without such adaptations, even evidence-based interventions may fail in practice.

Consistent with evidence from higher‑education teachers who reported benefits from short, routine‑compatible positive psychology practices (mindfulness and gratitude) facilitated by a trained practitioner [50], dancer interventions may achieve greater adherence and impact when designed as micro‑practices embedded within warm‑up/cool‑down or class transitions. Moreover, because positive psychology practices also improved professional relationships and classroom climate [50], brief positive psychology training for dance educators and choreographers could serve as a leverage point to shape supportive environments that mitigate performance anxiety and body‑image pressures. In line with this, Junge and Hauschild [5] recommended that future studies explore more flexible and less time-intensive interventions to address dancers’ mental health issues. Therefore, future interventions should be adapted to dancers’ unique schedules, even though theoretically effective methods risk poor engagement and limited ecological validity.

Among the included studies, only Walton et al. [57] employed an online, self-guided intervention without the involvement of an implementer, which demonstrated effectiveness in promoting dancers’ mental health. However, the mixed reception of online interventions highlights a dilemma for dance psychology: while digital tools increase accessibility, many dancers may still prefer the relational guidance of a facilitator [57]. This suggests that effectiveness depends not only on the content of the intervention but also on its delivery format and relational context, factors often underexplored in mental health research with performers. Consequently, future psychological interventions targeting pre-performance anxiety in dancers should prioritize the involvement of trained and qualified implementers, as they can provide personalized guidance, respond to participants’ individual needs in real time, and ensure proper technique and emotional support.

### Interventions for reducing eating disorder symptoms

Among the four studies targeting eating disorder symptoms in dancers [59, 62, 63, 66], only two reported successful outcomes [59, 62]. The researchers attributed the lack of significant improvements in dancers’ mental health through cognitive behavioral therapy to the absence of a control group, lack of randomization, and disruptions caused by the COVID-19 pandemic, which led to modifications in the intervention delivery, including sessions being canceled or led by less experienced instructors [63]. Gorrell et al. [66] identified two reasons for the lack of successful outcomes. First, the study was based on a relatively small sample (*N* = 23), which likely limited its statistical power and generalizability. Second, given the sensitive nature of the topic of their intervention, some students and parents may have opted out of participation because of confidentiality concerns, potentially introducing self-selection bias. Future studies should implement rigorous study designs, including RCTs with appropriate control groups, and ensure consistent delivery by qualified professionals.

Another factor potentially limiting intervention effectiveness is the mismatch between intervention design and the characteristics of the target population. On one hand, interventions adapted from sport (e.g., the Athlete Body Project [66]) may fail to capture the unique cultural and aesthetic pressures that shape dancers’ body image concerns. Unlike athletes, dancers’ concerns are closely tied to aesthetic ideals and professional identity, meaning that one-size-fits-all protocols transferred from athletic contexts are unlikely to be effective.

Similarly, Walton et al. [57] emphasized the importance of dance-specific psychological interventions, underscoring the need for tailored approaches to address issues such as disordered eating. When designing such interventions, it is also essential to account for the influence of dance discipline and gender, as these factors may shape both the prevalence and the expression of symptoms [67, 68]. Neglecting these contextual considerations risks producing interventions that are too generic or insufficiently sensitive to the realities of dance.

On the other hand, participant characteristics such as age and baseline risk also matter. For example, both Gorrell et al. [66] and Nordin-Bates et al. [63] recruited youth under 18, a group that may not have elevated risk for eating disorders [35] and may also lack the developmental maturity to fully benefit from techniques such as cognitive restructuring. Thus, null findings may reflect both cultural mismatches and developmental misalignments. These issues highlight the need for interventions that are both dance-specific and developmentally tailored, with careful attention to risk profile, intervention dosage, and delivery by appropriately trained facilitators.

Furthermore, most studies to date have focused exclusively on female ballet dancers [49, 60–64, 66], leaving gaps in understanding how male dancers or those in other genres (e.g., contemporary, hip-hop) experience and respond to interventions. This narrow evidence base not only limits generalizability but also perpetuates a one-dimensional view of dance.

Methodologically, few studies conducted stratified analyses by gender, age, or discipline, making it difficult to disentangle whether intervention effects vary across subgroups. Small sample sizes further constrain statistical power, raising the possibility that null findings reflect methodological shortcomings rather than genuine ineffectiveness. Future research should therefore examine whether interventions can be adapted to diverse dance populations while also considering how gender dynamics, dance discipline, and developmental stage intersect with broader cultural narratives of body image and performance.

### Psychological rehabilitation interventions for injured dancers

Surprisingly, the near absence of research on psychological rehabilitation in injured dancers is striking, particularly considering their high injury prevalence [69–71]. Given that both injured dancers may experience negative emotional responses during recovery [4, 9–12, 72], future research should prioritize well-designed RCTs and larger-scale interventions to evaluate the effectiveness of approaches such as meditation, goal setting, and self-talk. For example, Benoit-Piau et al. [72] demonstrated that motor imagery positively influences pain and function in injured dancers. However, none of the included studies applied motor imagery to the psychological rehabilitation of dancers. Future research should investigate its potential role in facilitating the psychological rehabilitation of injured dancers (e.g., by modulating pain perception). Developing and testing such interventions specifically within the dance population would strengthen the causal inferences, enhance the ecological validity of the findings, and ultimately provide evidence-based strategies to support dancers’ psychological well-being during rehabilitation.

### Methodological critique

Methodological weaknesses undermine the strength of the current evidence base. Of the 11 studies, only Walton et al. [57] met all five MMAT criteria and was rated as high quality. The reliance on small, non-randomized, short-term studies reflects a pattern of underinvestment in rigorous intervention research with dancers. For instance, Walton et al. [57] and Gorrell et al. [62, 66] implemented 3-week interventions. Harrison et al. [49] conducted a 6-week intervention, and Torres-McGehee et al. [59] reported a 4-week program. These limitations reduced both the generalizability of the findings and the strength of the causal inferences. Unless future research adopts more robust designs, the field risks perpetuating a cycle of weak evidence that cannot meaningfully inform practice.

Several studies addressed interventions for performance anxiety and emotional regulation. Interventions such as mindfulness, meditation, the Feldenkrais method, and solution-focused therapy showed promise; however, wide heterogeneity in the design, duration, delivery, and outcome measures limited comparability. Participant characteristics (e.g., age, dance type, and prior experience) were not consistently controlled, potentially influencing the responsiveness to interventions.

For eating disorder–related interventions, key methodological concerns included small samples (*N* = 19–40), short intervention durations (3–8 weeks), lack of long-term follow-up, and the adaptation of interventions originally designed for athletes rather than dancers. Gorrell et al. [66] reported null findings, which may reflect these design shortcomings rather than genuine ineffectiveness. Additionally, the sensitivity of topics such as eating behaviors could have introduced self-selection bias, as participants or their parents may have declined participation because of confidentiality concerns [66].

In the area of psychological rehabilitation following injuries, only a single-case study was identified [61]. While informative, case studies lack generalizability and cannot provide strong evidence of intervention effectiveness. Across this area, the absence of standardized outcome measures further limited the ability to compare psychological and functional outcomes.

A notable limitation of the current body of evidence is the inconsistent reporting of effect sizes. Of the 11 studies included in this review, only four studies [57, 58, 63, 66] provided standardized effect sizes, while the remaining studies reported only test statistics or descriptive results. This inconsistency limited the extent to which we could directly compare intervention effects across studies and precluded the possibility of conducting a meta-analysis. At the same time, this review makes an important contribution by drawing attention to this gap in the literature. Highlighting the lack of standardized effect size reporting underscores the need for future research on dancers to adopt more rigorous and transparent reporting practices. As recommended in the methodological literature [73], consistent reporting of effect sizes is essential for cumulative knowledge building, as it enables more meaningful synthesis, allows clearer comparisons across intervention approaches, and ultimately strengthens the evidence base for psychological interventions in dance.

Taken together, these methodological limitations underscore the need for future research to adopt more rigorous designs, including adequately powered RCTs, appropriate control groups, standardized and validated outcome measures, the reporting of effect sizes, and interventions specifically tailored to the unique psychological and cultural needs of dancers. Addressing these gaps is essential to building a robust evidence base that can meaningfully inform evidence-based practice for dancers’ mental health.

### Practical implications

This review is the first to focus exclusively on mental health interventions for dancers and to systematically categorize them by type. The findings yield several immediate implications for practitioners.

First, studies [49, 57] highlighted that time commitment posed a significant barrier for dancers, raising questions about the practicality of many existing interventions. Although shorter or more flexible formats have been suggested, few studies have systematically evaluated how interventions can be embedded into the realities of dancers’ demanding schedules. Evidence from other professional contexts supports the value of brief, culturally attuned positive psychology techniques, such as mindfulness and gratitude practices paired with core interventions, as feasible and effective when integrated into daily routines [50]. For psychological care to be effective in dance, it should be integrated into the culture of practice rather than compete with it. Collaboration between mental health professionals and dance educators, essential for achieving such integration, remains largely absent in both research and practice. Drawing on insights from the language-teacher study [50], brief interventions delivered by trained facilitators can support resilience, stress management, and positive interpersonal outcomes, highlighting opportunities to design ecologically valid interventions that fit dancers’ schedules without compromising efficacy.

Second, the limited effectiveness of certain approaches (e.g., cognitive behavioral therapy and the Athlete Body Project) suggests that interventions should be tailored to the specific characteristics of dancers rather than assumed to be transferable from athlete-focused protocols [63, 66]. Dancers’ experiences of body image, performance pressure, and identity differ in important ways from those of athletes, and interventions that fail to account for these differences risk reduced effectiveness. For example, recreational dancers may not experience the same intensity of body-related pressures as pre-professional or professional dancers, making certain eating-disorder-focused interventions less relevant or impactful in this subgroup. Conversely, professional dancers often face heightened scrutiny and perfectionistic expectations, which may exacerbate disordered eating symptoms [31] in ways that general athlete programs do not adequately address. This mismatch highlights a broader issue in the current evidence base: the tendency to adapt existing athlete-oriented protocols with minimal cultural or contextual modification. Without explicit attention to the unique psychosocial environment of dance, interventions are unlikely to achieve meaningful or sustainable outcomes.

Third, although research on dancers remains limited, emerging evidence suggests that guided motor imagery and other conservative approaches may support psychological rehabilitation after injuries [72]. Such techniques can help maintain dancers’ sense of connection to movement, reduce anxiety about returning to performance, and foster confidence during the recovery process. While these approaches hold promise, the current evidence base is preliminary, and their effectiveness has yet to be established through larger, well-controlled trials. Consequently, practitioners may cautiously incorporate guided motor imagery and related strategies as complementary tools, provided they are tailored to the unique physical and psychological demands of dance, while awaiting further empirical support.

Finally, following the context-driven perspectives of Schinke and Stambulova [74], interventions should be embedded within the broader realities of the dance environment, taking into account factors such as training climate, company culture, and the quality of instructor/choreographer–dancer relationships. These contextual elements shape how dancers perceive and engage with psychological support, and neglecting them may limit the applicability of otherwise effective approaches. For example, an intervention that aligns with a company’s rehearsal routines or acknowledges the hierarchical dynamics between dancers and choreographers is more likely to be accepted and sustained.

The effectiveness of interventions can be further enhanced when delivered by trained practitioners who provide technical guidance, monitor dancers’ progress, adapt strategies to individual needs, and foster trust and safety [47]. Positive psychology techniques can be translated into brief, low-burden routines embedded within existing dance practice, such as a two-minute guided breath-and-gratitude check-in at the start of class, a short “savoring” reflection at the end of rehearsal, or weekly peer-based strengths-spotting exercises that normalize self-compassion and mutual support. Like teachers with heavy workloads, dancers have limited discretionary time; positive psychology micro-practices, such as brief mindfulness, gratitude journaling, and strength-spotting, can be delivered in two to five minutes and anchored to warm-up, cool-down, or transitional moments, enhancing adherence while supporting resilience and a positive climate. Introduced and periodically refreshed by trained practitioners, then maintained by dance educators or company staff, these micro-practices offer a feasible, sustainable, and ecologically valid approach to integrating mental health support into daily dance culture.

Notably, among the 11 interventions included in this review, only the case reported by Roncaglia [61] explicitly incorporated co-production with the dancer. In that intervention, an open, authentic, and collaborative space was established, and both process and performance goals were jointly set with the dancer, who was actively involved in self-reflection and development throughout the program. The remaining 10 interventions were researcher-led, with no reported engagement or consultation with dance community members. This highlights a critical direction for future intervention design: psychological programs should be co-produced with dancers, educators, and company staff to ensure cultural relevance, ownership, and ecological validity. Co-creation not only strengthens engagement and adherence but also transforms interventions from externally imposed programs into shared, community-driven practices that align with the lived realities of the dance environment.

### Study limitations

This review has several limitations. First, the reliance on published literature may have introduced publication bias, as we excluded relevant gray literature. Second, this review synthesized evidence from a relatively small number of studies (*K* = 11), and the limited availability of high-quality RCTs (only two studies, [49, 62]) reduced the strength of the causal inferences that could be made.

Third, due to the limited number of relevant studies, two included studies [57, 58] examined performing artists more broadly, encompassing musicians, actors/actresses, and dancers, without providing separate analyses for the dancer subgroup. As a result, it remains uncertain to what extent these findings reflect dancers’ unique experiences or responses. The inclusion of such mixed-population studies, while necessary to capture the sparse evidence base, may limit the specificity and interpretability of conclusions regarding dance-focused psychological interventions.

Fourth, in relation to psychological rehabilitation for injuries, only one single-case study [61] was identified, which stands in contrast to the high injury rates among dancers [69–71]. This highlights a critical gap in the literature and underscores the need for future research employing well-designed RCTs and larger-scale interventions to evaluate psychological approaches tailored to dancers. Finally, while we used the MMAT [52] to assess the methodological quality of the included studies, one of the studies was a single-case study [61] that was not compatible with this appraisal tool, potentially affecting the overall rigor of this review.

### Recommendations for future research

Based on the findings and limitations of this review, we propose several directions for future research on psychological interventions for dancers. First, research should test brief mindfulness practices, online or self-guided programs, and other delivery methods, while systematically evaluating the minimum effective dosage and optimal frequency. Second, more studies are needed on techniques such as goal setting, self-talk, and motor imagery for injured dancers, with evaluations of both psychological and functional outcomes.

Third, building on qualitative design templates, future work could include focus groups with dancers and educators to explore the acceptability, cultural fit, and perceived benefits of positive psychology–informed micro-practices. Insights from these focus groups could then inform small quasi-experimental pilot studies comparing “business-as-usual” approaches with embedded positive psychology micro-practices, assessing outcomes such as performance/competitive anxiety, self-compassion, body appreciation, and adherence. Echoing positive psychology work with East Asian higher-education teachers, future dancer interventions should be culturally and institutionally tailored, with co-design input from dancers and staff to ensure ecological fit. Lastly, future research should investigate the feasibility of integrating psychological support into physical rehabilitation programs, which could improve dancers’ overall recovery experiences.

## Conclusion

This review synthesized existing psychological interventions aimed at improving dancers’ mental health. Although various interventions have been implemented on dancers to reduce performance anxiety, eating disorder symptoms, and negative psychological responses following dance injuries as well as to aid in the management of emotional regulation, their effectiveness remains inconsistent as a result of the variety of intervention types, the duration of interventions, and participants’ time constraints. Future studies should focus on developing targeted, evidence-based interventions that consider the unique demands of the dance profession.

## Supplementary Information

Supplementary Material 1.

Supplementary Material 2.

## Data Availability

Data available on request from the authors. The data that support the findings of this study are available from the corresponding author upon reasonable request.
