# Supplementary material for: Psychological interventions for dancers’ mental health: a systematic review
Source: BMC Psychol. 2025 Nov 22;13:1392. doi: 10.1186/s40359-025-03724-7 (PMC12750959; doi:10.1186/s40359-025-03724-7)
Supplement: Supplementary file 2 — Supplementary Material 2. [file 40359_2025_3724_MOESM2_ESM.docx]

Supplementary Material 2. *MMAT Quality Appraisal of Studies with Comments (Chronological Order)*

| Author(s) | Screening questions | | Qualitative | | | | | Quantitative (randomized) | | | | | Quantitative  (non-randomized) | | | | | Mixed methods | | | | | Comments |
| --- | --- | --- | --- | --- | --- | --- | --- | --- | --- | --- | --- | --- | --- | --- | --- | --- | --- | --- | --- | --- | --- | --- | --- |
| Walton et al. (2025) | ✓ | ✓ |  |  |  |  |  |  |  |  |  |  |  |  |  |  |  | ✓ | ✓ | ✓ | ✓ | ✓ | - This study meets all the criteria for MMAT assessment. |
| Harrison et al. (2025) | ✓ | ✓ |  |  |  |  |  | ✓ | ✓ | ✓ | – | ✓ |  |  |  |  |  |  |  |  |  |  | - The blinding of outcome assessors is not specified in the study. - Harrison et al. (2024) considered their study design to be a non-randomized controlled trial, while the authors of this paper believe it to be a pre-post test design. |
| Chen et al. (2024) | ✓ | ✓ |  |  |  |  |  |  |  |  |  |  | ✓ | ✓ | X | ✓ | – |  |  |  |  |  | - Missing participant demographic information. - Missing information on the duration of the single intervention. |
| Gorrell et al. (2024) | ✓ | ✓ |  |  |  |  |  |  |  |  |  |  | X | X | ✓ | X | ✓ |  |  |  |  |  | - The study did not include the full sample, as some children and parents did not provide consent, limiting its ability to fully represent the target population. - The study failed to use an evidence-based acceptability measurement tool. - The study did not control for potential confounding variables, such as systematic differences between children who provided consent and those who did not, which may affect the external validity of the study. |
| Lubert et al. (2023) | ✓ | ✓ |  |  |  |  |  |  |  |  |  |  | X | X | X | ✓ | X |  |  |  |  |  | - Due to the absence of a control group, the causal effects of the intervention cannot be clearly determined, which presents certain limitations in data interpretation. - The design of personalized interventions is difficult to replicate, which affects the reproducibility of the study. - The study failed to include all initially selected artists, and the uneven distribution of participants across music, dance, and theater affected its representativeness. - The study mentions a small sample size, with a statistical power of only 70%, indicating that the quantitative component of the study may have statistical limitations. |
| Nordin-Bates et al. (2023) | ✓ | ✓ |  |  |  |  |  |  |  |  |  |  | ✓ | ✓ | ✓ | ✓ | X |  |  |  |  |  | - One nutrition workshop was cancelled due to the COVID-19 pandemic. - 60% of the cognitive behavioral therapy workshops were taught by an instructor with less experience. |
| Roncaglia (2023) | ✓ | ✓ |  |  |  |  |  |  |  |  |  |  |  |  |  |  |  |  |  |  |  |  | - This is a single case study. |
| Stackpole & Quiroga-Garza (2023) | ✓ | ✓ |  |  |  |  |  |  |  |  |  |  | ✓ | ✓ | X | ✓ | ✓ |  |  |  |  |  | - This study has a small sample. - The proportion of female participants was too high. - The age range of the participants was wide. |
| Wallman-Jones et al. (2023) | ✓ | ✓ | ✓ | X | X | ✓ | ✓ |  |  |  |  |  |  |  |  |  |  |  |  |  |  |  | - The control group did not receive additional interventions (such as an equivalent amount of dance training), which may lead to bias due to training effects or fatigue effects. - The sample size was small and only constituted a feasibility study, limiting statistical power. - There was a significant age difference between the intervention and control groups. |
| Gorrell et al. (2021) | ✓ | ✓ |  |  |  |  |  | ✓ | X | X | X | ✓ |  |  |  |  |  |  |  |  |  |  | - A strict wait-list control group was not established. - Adequate control variables and post-intervention follow-up were not provided. - The study did not use an empirically support acceptability measurement tool. - The focus group interviews were conducted by the researcher, which may have led to social desirability bias. - The dietary restraint measurement in the T1 assessment had low reliability. - The small sample size (pilot study) limited the statistical power. |
| Torres-McGehee et al. (2011) | ✓ | ✓ |  |  |  |  |  |  |  |  |  |  | ✓ | ✓ | X | X | ✓ |  |  |  |  |  | - The sample was limited to participants from two Tier 1 institutions in the southeastern United States, restricting the generalizability of the findings. - Participants may have provided socially desirable responses out of concern that the data would be shared with their coaches, affecting the reliability and authenticity of the data. |

*Note.* Q1: Are there clear research questions? Q2: Do the collected data allow addressing the research questions?

‘✓’ means that the criterion is met. ‘X’ means that the criterion is not met. ‘–’ means that there is not enough information in the paper to judge if the criterion is met or not. Five criteria for the qualitative studies are as follows: 1. Is the qualitative approach appropriate to answer the research question? 2. Are the qualitative data collection methods adequate to address the research question? 3. Are the findings adequately derived from the data? 4. Is the interpretation of results sufficiently substantiated by data? 5. Is there coherence between qualitative data sources, collection, analysis and interpretation?

Five criteria for the quantitative (randomized) studies are as follows: 1. Is randomization appropriately performed? 2. Are the groups comparable at baseline? 3. Are there complete outcome data? 4. Are outcome assessors blinded to the intervention provided? 5. Did the participants adhere to the assigned intervention?

Five criteria for the quantitative (non-randomized) studies are as follows: 1. Are the participants representative of the target population? 2. Are the measurements appropriate in relation to both the outcome and the intervention (or exposure)? 3. Are the outcome data complete? 4. Are the confounders addressed in the study design and analysis? 5. Was the intervention administered (or exposure occurred) as intended during the study period?

Five criteria for the mixed methods studies are as follows: 1. Is there an adequate rationale for using a mixed methods design to address the research question? 2. Are the different components of the study effectively integrated to answer the research question? 3. Are the outputs of the integration of qualitative and quantitative components adequately interpreted? 4. Are divergences and inconsistencies between quantitative and qualitative results adequately addressed? 5. Do the different components of the study adhere to the quality criteria of each tradition of the methods involved?

Since Quantitative (descriptive) studies were not included, they are not displayed in the table.
